# Identification of prothymosin alpha (PTMA) as a biomarker for esophageal squamous cell carcinoma (ESCC) by label-free quantitative proteomics and Quantitative Dot Blot (QDB)

**DOI:** 10.1186/s12014-019-9232-6

**Published:** 2019-04-05

**Authors:** Yanping Zhu, Xiaoying Qi, Cuicui Yu, Shoujun Yu, Chao Zhang, Yuan Zhang, Xiuxiu Liu, Yuxue Xu, Chunhua Yang, Wenguo Jiang, Geng Tian, Xuri Li, Jonas Bergquist, Jiandi Zhang, Lei Wang, Jia Mi

**Affiliations:** 1 Precision Medicine Research Center, Binzhou Medical University, No. 346 Guanhai Rd., Laishan District, Yantai, 264003 Shandong Province People’s Republic of China; 2 Department of Anesthesiology, The Affiliated Yantai Yuhuangding Hospital of Qing Dao University, No. 20 Yudong Rd., Zhifu District, Yantai, S264009 Shandong People’s Republic of China; 3 Department of Ultrasound, Yantai Affiliated Hospital of Binzhou Medical University, No. 717 Jinfu Rd., Muping District, Binzhou, 264100 Shandong Province People’s Republic of China; 4 State Key Laboratory of Ophthalmology, Zhongshan Ophthalmic Center, Sun Yat-Sen University, Guangzhou, 510060 People’s Republic of China; 5 Department of Chemistry, BMC, Uppsala University, PO Box 599, Husargatan 3, 75124 Uppsala, Sweden; 6 Yantai Zestern Biotechnique Co. LTD, 39 Keji Ave. Bioasis, Yantai, People’s Republic of China; 7 Department of Thoracic Surgery, The First Affiliated Hospital of Harbin Medical University, No. 23, Youzheng Street, Nangang District, Harbin, 150000 Heilongjiang Province People’s Republic of China

**Keywords:** Esophageal squamous cell carcinoma (ESCC), Label-free quantitative proteomics, Prothymosin alpha (PTMA), Quantitative Dot Blot (QDB)

## Abstract

**Background:**

Esophageal cancer (EC) is one of the malignant tumors with a poor prognosis. The early stage of EC is asymptomatic, so identification of cancer biomarkers is important for early detection and clinical practice.

**Methods:**

In this study, we compared the protein expression profiles in esophageal squamous cell carcinoma (ESCC) tissues and adjacent normal esophageal tissues from five patients through high-resolution label-free mass spectrometry. Through bioinformatics analysis, we found the differentially expressed proteins of ESCC. To perform the rapid identification of biomarkers, we adopted a high-throughput protein identification technique of Quantitative Dot Blot (QDB). Meanwhile, the QDB results were verified by classical immunohistochemistry.

**Results:**

In total 2297 proteins were identified, out of which 308 proteins were differentially expressed between ESCC tissues and normal tissues. By bioinformatics analysis, the four up-regulated proteins (PTMA, PAK2, PPP1CA, HMGB2) and the five down-regulated proteins (Caveolin, Integrin beta-1, Collagen alpha-2(VI), Leiomodin-1 and Vinculin) were selected and validated in ESCC by Western Blot. Furthermore, we performed the QDB and IHC analysis in 64 patients and 117 patients, respectively. The PTMA expression was up-regulated gradually along the progression of ESCC, and the PTMA expression ratio between tumor and adjacent normal tissue was significantly increased along with the progression. Therefore, we suggest that PTMA might be a potential candidate biomarker for ESCC.

**Conclusion:**

In this study, label-free quantitative proteomics combined with QDB revealed that PTMA expression was up-regulated in ESCC tissues, and PTMA might be a potential candidate for ESCC. Since Western Blot cannot achieve rapid and high-throughput screening of mass spectrometry results, the emergence of QDB meets this demand and provides an effective method for the identification of biomarkers.

## Introduction

Esophageal cancer (EC) is one of the malignant tumors with a 5-year survival incidence of 20.9% [1, 2]. EC is ranked as the eighth most common malignant tumor with the sixth highest mortality rate worldwide. There are two histological subtypes of EC: esophageal squamous cell carcinoma (ESCC) and esophageal adeno carcinoma (EAC). ESCC often occurs in the top or middle of the esophagus, and starts in the flat thin cells that make up the lining of the esophagus. Meanwhile, EAC is most common in the lower portion of the esophagus, and starts in the glandular cells that are responsible for the production of fluids such as mucus. China is a high-risk area for EC, and more than 90% of cases are esophageal squamous cell carcinoma (ESCC) [3–5]. Moreover, most of the patients exhibit locally advanced or metastatic EC at the time of being diagnosed [6, 7]. Therefore, it is urgent to discover biomarkers for early clinical diagnosis to improve survival.

Esophageal cancer biomarkers have been found in saliva, blood, and urine. Sedighi et al. showed that the serum level of Matric metalloproteinase (MMP)-13 in ESCC patients were significantly higher than in the control group, and suggested that the MMP-13 was associated with increasing ESCC invasion, lymph node involvement and decreased survival rates [8]. In saliva, the miRNAs (miR-10b*, miR-144 and miR-451) were identified up-regulated expression in EC, which possessed discriminatory ability of detecting EC [9]. Although these biomarkers contribute to the early diagnosis and prognosis of EC, the EC biomarker is still in the stage of exploration and verification, with limitations of specificity and low sensitivity.

Proteomic technologies have been applied to understand tumor pathogenesis, and to discover novel targets for cancer therapy or prognosis. Combining MS-based proteomic data with integrative bioinformatics can predict protein signal network and identify more clinical relevant molecules [10–12]. To date, quantitative proteomic methods have been applied in the study of various cancer, such as breast cancer, lung cancer, pancreatic cancer and gastric cancer [13]. Mass spectrometric identification of differentially expressed proteins has been a highly successful approach for finding novel cancer-specific biomarkers [14]. For more than a decade, attempts have been made to uncover valid biomarkers for the diagnosis of EC. Currently, various molecules have been identified as closely correlated with ESCC, such as transgelin (TAGLN) and proteasome activator 28-beta subunit (PA28β) [15], pituitary tumor transforming gene (PTTG) [6], transglutaminase 3 (TGM) by proteomics [2]. However, the number of proteins identified was limited in these studies and they did not provide validation of the suggested biomarkers. Therefore, it is still necessary to perform further in-depth proteomics to explore novel candidate biomarkers for EC, and to validate the findings with orthogonal techniques.

Differential proteins obtained from mass spectrometry are commonly identified by Western Blot. However, it couldn’t meet the requirements for high-throughput analysis, due to the complicated processing steps and the requirements for large amount of total protein. Recently, Quantitative Dot Blot (QDB) technology developed by our team achieves high-throughput quantitative detection with the same principle of traditional Western Blot. In addition, QDB technology has the advantages of less sample consumption, short time consumption and low cost [16]. The experiment has been successfully applied to the detection of biomarker of papillary thyroid carcinoma. With its accuracy and reliability, the QDB is a very effective method for protein detection.

The aim of this study was to investigate the protein expression profiles in ESCC tissues and adjacent normal esophageal tissues with a label-free quantitative proteomics approach through nano-liquid chromatography coupled with tandem mass spectrometry (Nano-LC–MS/MS). The differentially expressed proteins were selected and their expression trends were validated in ESCC by Western Blot, then high-throughput protein screening was achieved by QDB, and the results of QDB were verified by classical IHC experiment. This research provides a new methodological strategy for validation and identification ESCC biomarkers by combining quantitative proteomic with QDB.

## Materials and methods

### Tissue samples

The five patients for LC/MS analysis were all male, with the average age of 61. Samples of ESCC tissues and adjacent normal esophageal tissues were taken for mass spectrometry analysis. The 64 pairs of matched ESCC and adjacent normal tissue samples for QDB were based on a clear pathological diagnosis, which included 35 men and 29 women, with an age range of 46–73 years (mean 61 years). The above samples were obtained at the Affiliated Yantai Hospital of Binzhou Medical University. All data were obtained from patient medical records. All specimens were quickly rinsed and then frozen immediately in liquid nitrogen and then stored at − 80 °C until further processing. The tissue microarrays (TMA) (ES701 and ES1922) for immunohistochemistry analysis were purchased from the alenabio company, the total sample size reached 117 pairs after removing duplicates in two arrays (n = 14). This study was approved by the Human Research Ethics Committee of Binzhou Medical University.

### Reagents

Rabbit anti-PPP1CA (CSB-PA030161) and rabbit anti-PAK2 (CSB-PA622641DSR1HU) were purchased from CUSABIO (Wuhan, China). Rabbit anti-PTMA (YN2871) and rabbit anti-HMGB-2 (YT2187) were purchased from ImmunoWay Biotechnology Company (USA). The antibody of Caveolin (AF0126), Integrin beta-1 (AF5379), Collagen alpha-2(VI) (DF3552), Leiomodin-1 (DF12160) and Vinculin (AF5122) were purchased from Affinity Biosciences (USA). Mouse anti-GAPDH monoclonal antibody (sc-32233) was purchased from Santa Cruz Biotechnology (Dallas, TX, USA). Goat anti-rabbit (127,760) and goat anti-mouse (124,227) secondary antibodies were purchased from ZSGB-BIO (Beijing, China).

### Sample preparation

The 5 pairs of clinical samples were homogenized and broken with lysis buffer containing 9 M Urea, 20 mM HEPES, and protease inhibitor cocktail. The samples were centrifuged at 12,000×*g* for 10 min at 4 °C and supernatants retained. Then 20 μg of total protein were digested using the way of in-solution digestion. Firstly, the samples were reduced with 50 mM dithiothreitol (DTT) at 50 °C for 15 min, then alkylated with 50 mM iodoacetamide (IAA) for 15 min in darkness, and then diluted 4 times with digestion buffer (50 mM NH_4_HCO_3_, pH 8.0). The proteins were digested by Trypsin with a final concentration of 5% (w/w), then incubated at 37 °C overnight. The reaction was stopped by diluting the sample 1:1 with trifluoroacetic acid (TFA) in acetonitrile (ACN) and Milli-Q water (1/5/94 v/v). Finally, peptides were desalted using Pierce C18 Spin Columns and dried completely in a vacuum centrifuge.

### LC–MS/MS

The peptides were dissolved in 20 μL 0.5% TFA in 5% ACN and analyzed using QExactive Plus Orbitrap™ mass spectrometer (Thermo Fisher Scientific, Bremen, Germany) coupled with the liquid chromatography system (EASY-nLC 1000, Thermo Fisher Scientific, Bremen, Germany). A 85-min LC gradient was applied, with a binary mobile phase system of buffer A (0.1% formic acid) and buffer B (80% acetonitrile with 0.1% formic acid) at a flow rate of 250 nL/min. In MS analysis, peptides were loaded onto the 2 cm EASY-column precolumn (1D 100 μm, 5 μm, C18, Thermo Fisher Scientific), and eluted at a 10 cm EASY-column analytical column (1D 75 μm, 3 μm, C18, Thermo Fisher Scientific). For information data dependent analysis (DDA), full scan MS spectra were executed in the m/z range 150–2000 at a resolution of 70,000. The peptides elution was performed with a linear gradient from 4 to 100% ACN at the speed 250 nL/min in 90 min. Then the top 10 precursors were dissociated into fragmentation spectra by high collision dissociation (HCD) in positive ion mode.

### Proteomic data processing

The acquired data were analyzed by using Maxquant (version 1.5.0.1) against the UniProt Homo sapiens database. The searching parameters were set as maximum 10 and 5 ppm error tolerance for the survey scan and MS/MS analysis, respectively. The enzyme was trypsin, and two missed cuts were allowed. The max number of modifications per peptide is 5. Using the Label-free quantification (LFQ), the LFQ minimum ratio count was set to 2. The FDR (false discovery rate) was set to 1% for the peptide spectrum matches (PSMs) and protein quantitation. Gene ontology and protein class analysis were performed with the PANTHER system (http://pantherdb.org/). Meanwhile, the heat map of significantly different proteins was screened by using Morpheus (https://software.broadinstitute.org/morpheus). The protein–protein interaction analysis of the differently expressed proteins was performed by STRING (https://string-db.org/).

### Western blot (WB)

Tissues lysates were prepared by using highly efficient RIPA lysis buffer including PMSF (Phenylmethanesulfonyl fluoride). The total proteins were quantified by BCA protein assay kit and then separated by sodium dodesyl sulphate–polyacrylamide gel electrophoresis (SDS-PAGE). Equal amounts of protein were separated by 6%, 15% and 12% SDS-PAGE, respectively. Subsequently, proteins were transferred to a PVDF membrane and then blocked with TBS (pH 7.4) containing 0.05% Tween 20 and 5% nonfat milk. Next, the membranes were incubated with rabbit anti-PTMA (1:1000), rabbit anti-HMGB-2 (1:500), rabbit anti- PPP1CA (1:1000), rabbit anti-PAK2 (1:1000), and mouse anti-GAPDH (1:1000) antibodies at 4 °C overnight, respectively. The other five antibodies (Caveolin, Integrin beta-1, Collagen alpha-2(VI), Leiomodin-1 and Vinculin) were diluted in a ratio of 1:200. After washing, membranes were incubated with goat anti-rabbit (1:2000) and goat anti-mouse (1:2000) secondary antibodies at room temperature for 1 h. The ECL system was used to detect protein expression.

### QDB

The total proteins were quantified by BCA protein assay kit and then validated by Quantitative Dot Blot (QDB). Firstly, we determined the linear range of PTMA of the QDB analysis, through the testing of series of concentrations including 0, 0.25, 0.5, 1, 2 and 4 μg/μL. After that, equal amounts of protein were loaded. The sample was incubated at 37 °C for 15 min or until the membrane was completely dried. To block the plate, the QDB plate was dipped in 20% methanol. The plate was then washed with TBST, followed by 5% fat-free milk under constant shaking at room temperature for 1 h. After washing with TBST, the QDB plate was placed in a 96 well plate and 100 μL of primary antibodies was separately added to each individual well and shaken overnight at 4 °C. After washing the QDB plate, 100 μL of the secondary antibody was added to each well and incubated for 1 h at room temperature with shaking. Samples were washed with TBST and detected with the ECL substrate using a Tecan Infiniti 200 pro microplate reader. For each sample, a triplicate measurement was performed, and the average value was obtained. The relative quantitation of each PTMA protein in the lysates was then calculated.

### Immunohistochemistry (IHC)

The PTMA expression was detected by IHC in tissue microarrays (TMA) (ES701, ES1922). Firstly, the tissue microarrays were heated at 60 °C for 30 min, then deparaffinized and hydrated with xylol and gradient alcohol, respectively. Next, the antigen retrieval was accomplished by boiling the TMAs for 10 min in citrate buffer (0.01 M, pH 6.0). After cooling at room temperature, the microarrays were treated with 3% hydrogen peroxide for 30 min at 37 °C. The samples were blocked with bovine serum albumin for 30 min at 37 °C, then the PTMA antibody (YN2871, ImmunoWay; dilution 1:50) were incubated overnight at 4 °C in a moist chamber. After using the Histostain-SP (Streptavidin–Peroxidase) kit (SP-0023) as the secondary antibody following the recommendation from the manufacture, operation manual, the samples were washed with PBS (0.01 M, pH 7.2–7.4). Finally, the immunoreactivity was detected by DAB Horseradish Peroxidase Color Development Kit.

### Statistics analysis

The WB data was analyzed by means and standard deviation for four independent experiments. The other data was compared between esophageal cancer tissues and adjacent normal esophageal tissues using the two-tailed paired Student’s *t* test. All statistical analyses were performed by using the statistical software SPSS v20.0 (Chicago, Illinois, USA). *P* < 0.05 was considered statistically significant.

## Results

### Identification of differently expressed proteins

**Fig. 1:**
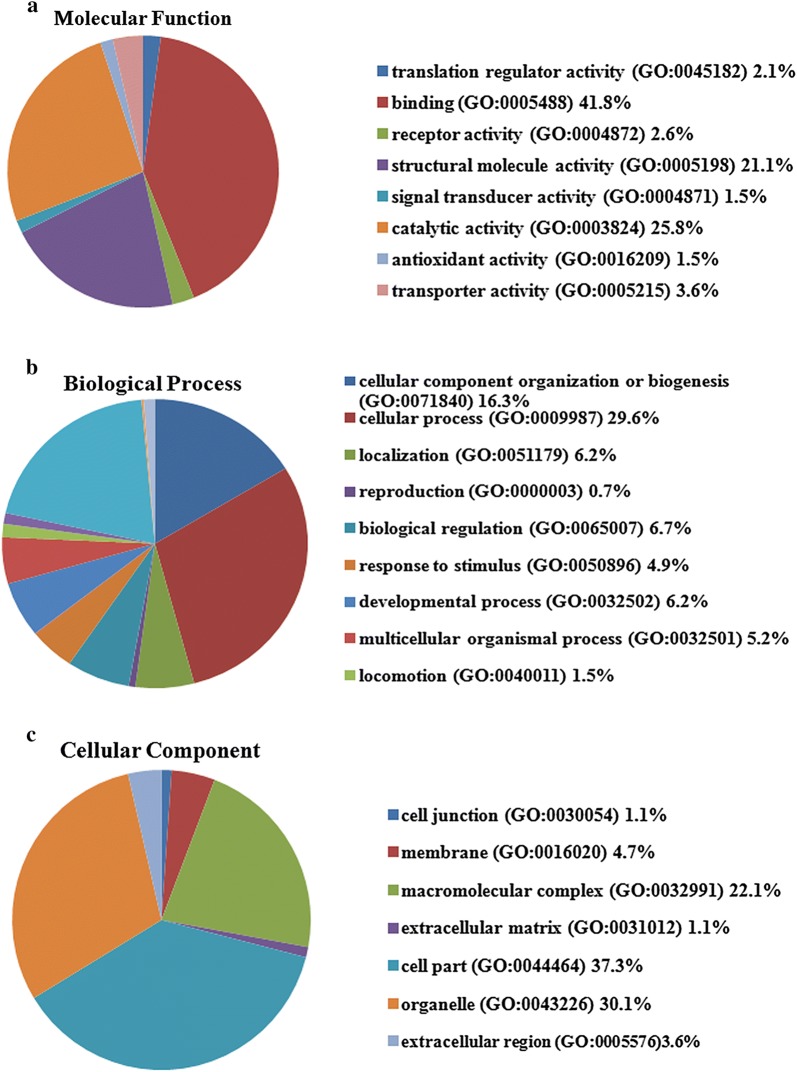
Classification of identified proteins by gene ontology based on their **a** molecular function, **b** biological process and **c** cellular component. The analysis of proteins were performed via the PANTHER (http://pantherdb.org/)

The clinical information of the five patients was summarized in Table 1. The five pairs of cancer tissues and adjacent normal tissues were analyzed by label-free mass spectrometry. Total 2297 proteins were identified and 308 proteins with significant differences were selected. Among these proteins, 102 proteins were expressed only in ESCC tissues (Table 2), 155 proteins were significantly up-regulated (Table 3) and 40 proteins were down-regulated in ESCC tissues (Table 4) (*P* < 0.05). Using the PANTHER classification system, we analyzed the biological significance of these proteins including the cellular component, molecular function and biological process (Fig. 1). The majority of proteins belonged to cell part proteins (37.3%) and organelle proteins (30.1%), possessed the ability of binding (41.8%) and catalytic activity (25.8%), and involved in the cellular process (29.6%), metabolic process (20.2%), cellular component organization or biogenesis (16.3%).

**Table 1:** The clinical features of ESCC patients for mass spectrometry

No.	Gender	Age	Organ/anatomic site	Grade	TNM
1	Male	69	Mid-thoracic esophagus	II	T2N0MO
2	Male	61	esophagus	I	T1N0M0
3	Male	59	Middle-lower esophagus	II	T1N0M0
4	Male	52	Mid-thoracic esophagus	III	T3N0M0
5	Male	64	Middle segment of esophagus	II	T2N1M1

**Table 2:** List of 102 proteins that were uniquely identified in ESCC tissues

Protein IDs	Protein names
P30050	60S ribosomal protein L12
P25788	Proteasome subunit alpha type-3
Q15254	Prothymosin alpha
P12956	X-ray repair cross-complementing protein 6
O15371	Eukaryotic translation initiation factor 3 subunit D
Q59FF0	Staphylococcal nuclease domain-containing protein 1
Q06323	Proteasome activator complex subunit 1
Q15366	Poly(rC)-binding protein 2;Poly(rC)-binding protein 3
Q99729	Heterogeneous nuclear ribonucleoprotein A/B
P62273	40S ribosomal protein S29
O15144	Actin-related protein 2/3 complex subunit 2
Q07955	Serine/arginine-rich splicing factor 1
Q13838	Spliceosome RNA helicase DDX39B
Q14666	Keratin, type I cytoskeletal 17
P00491	Purine nucleoside phosphorylase
P13667	Protein disulfide-isomerase A4
P49755	Transmembrane emp24 domain-containing protein 10
P34932	Heat shock 70 kDa protein 4
P62750	60S ribosomal protein L23a
Q9BRL6	Serine/arginine-rich splicing factor 2
P26583	High mobility group protein B2
O60716	Catenin delta-1
Q13151	Heterogeneous nuclear ribonucleoprotein A0
P62244	40S ribosomal protein S15a
Q8TBK5	60S ribosomal protein L6
P39656	Dolichyl-diphosphooligosaccharide–protein glycosyltransferase 48 kDa subunit
Q53GA7	Tubulin alpha-1C chain
Q92598	Heat shock protein 105 kDa
Q92928	Ras-related protein Rab-1B
Q59F66	Probable ATP-dependent RNA helicase DDX17
P46782	40S ribosomal protein S5
P78417	Glutathione S-transferase omega-1
P23526	Adenosylhomocysteinase
P62081	40S ribosomal protein S7
P11413	Glucose-6-phosphate 1-dehydrogenase
P67809	Nuclease-sensitive element-binding protein 1
Q08211	ATP-dependent RNA helicase A
P17980	26S protease regulatory subunit 6A
Q59EG8	26S proteasome non-ATPase regulatory subunit 2
P27695	DNA-(apurinic or apyrimidinic site) lyase, mitochondrial
P61019	Ras-related protein Rab-2A
P28066	Proteasome subunit alpha type
P49588	Alanine–tRNA ligase, cytoplasmic
O14818	Proteasome subunit alpha type
Q8NB80	Serine/arginine-rich splicing factor 7
Q86UE4	Protein LYRIC
P83731	60S ribosomal protein L24
B4DDM6	Mitotic checkpoint protein BUB3
P20618	Proteasome subunit beta type
P31942	Heterogeneous nuclear ribonucleoprotein H3
Q13177	Serine/threonine-protein kinase PAK 2
P53621	Coatomer subunit alpha;Xenin;Proxenin
Q04760	Lactoylglutathione lyase
Q99439	Calponin;Calponin-2
P62266	40S ribosomal protein S23
P62857	40S ribosomal protein S28
O43852	Calumenin
Q567R6	Single-stranded DNA-binding protein
P22234	Multifunctional protein ADE2
P62195	26S protease regulatory subunit 8
P98179	RNA-binding protein 3
P46781	40S ribosomal protein S9
Q96FW1	Ubiquitin thioesterase OTUB1
O14979	Heterogeneous nuclear ribonucleoprotein D-like
P51571	Translocon-associated protein subunit delta
P05455	Lupus La protein
Q96AE4	Far upstream element-binding protein 1
P17844	Probable ATP-dependent RNA helicase DDX5
P52597	Heterogeneous nuclear ribonucleoprotein F
P60866	40S ribosomal protein S20
Q13148	TAR DNA-binding protein 43
P62136	Serine/threonine-protein phosphatase PP1-alpha catalytic subunit
P07602	Prosaposin
P62633	Cellular nucleic acid-binding protein
Q6FI03	Ras GTPase-activating protein-binding protein 1
P51572	B-cell receptor-associated protein 31
P27635	60S ribosomal protein L10
Q09028	Histone-binding protein RBBP4
Q9UMS4	Pre-mRNA-processing factor 19
P62318	Small nuclear ribonucleoprotein Sm D3
Q15056	Eukaryotic translation initiation factor 4H
P38159	RNA-binding motif protein, X chromosome
Q1KMD3	Heterogeneous nuclear ribonucleoprotein U-like protein 2
P17987	T-complex protein 1 subunit alpha
Q13263	Transcription intermediary factor 1-beta
P29590	Protein PML
Q92499	ATP-dependent RNA helicase DDX1
P51858	Hepatoma-derived growth factor
P60468	Protein transport protein Sec61 subunit beta
Q13185	Chromobox protein homolog 3
P55209	Nucleosome assembly protein 1-like 1
P50454	Serpin H1
P42704	Leucine-rich PPR motif-containing protein, mitochondrial
P61204	ADP-ribosylation factor 1;ADP-ribosylation factor 3
Q9HB71	Calcyclin-binding protein
P11166	Solute carrier family 2, facilitated glucose transporter member 1
Q9Y265	RuvB-like 1
P62807	Histone H2B
Q9UK76	Hematological and neurological expressed 1 protein
P12004	Proliferating cell nuclear antigen
P43243	Matrin-3
P62333	26S protease regulatory subunit 10B

**Table 3:** List of 155 proteins that were overexpressed in ESCC tissues

IDs	Log ratio	*P* value	Protein names
P60842	7.814	0.000	Eukaryotic initiation factor 4A-I
P23396	6.277	0.000	40S ribosomal protein S3
P52272	7.623	0.000	Heterogeneous nuclear ribonucleoprotein M
P43686	10.195	0.000	26S protease regulatory subunit 6B
P14866	8.871	0.000	Heterogeneous nuclear ribonucleoprotein L
P53675	5.484	0.001	Clathrin heavy chain;Clathrin heavy chain 1
P84090	11.171	0.001	Enhancer of rudimentary homolog
P22392	12.881	0.001	Nucleoside diphosphate kinase
Q01105	7.330	0.001	Protein SET;Protein SETSIP
P84103	7.084	0.001	Serine/arginine-rich splicing factor 3
P07900	9.462	0.001	Heat shock protein HSP 90-alpha
Q01518	2.076	0.001	Adenylyl cyclase-associated protein
Q15233	22.489	0.001	Non-POU domain-containing octamer-binding protein
P51149	7.249	0.001	Ras-related protein Rab-7a
Q05CK9	9.797	0.001	Heterogeneous nuclear ribonucleoprotein Q
P10809	9.235	0.001	60 kDa heat shock protein, mitochondrial
P68371	1.935	0.001	Tubulin beta-4B chain
P37802	3.333	0.001	Transgelin-2
P62826	6.962	0.002	GTP-binding nuclear protein Ran
P25398	4.816	0.002	40S ribosomal protein S12
P57723	4.611	0.002	Poly(rC)-binding protein 1
Q12906	28.577	0.002	Interleukin enhancer-binding factor 3
P08865	5.309	0.002	40S ribosomal protein SA
P63244	6.237	0.002	Guanine nucleotide-binding protein subunit beta-2-like 1
P14314	14.510	0.002	Glucosidase 2 subunit beta
P60900	9.105	0.002	Proteasome subunit alpha type
P06748	12.711	0.002	Nucleophosmin
P05388	8.012	0.002	60S acidic ribosomal protein P0
P46940	3.595	0.003	Ras GTPase-activating-like protein IQGAP1
P61978	10.444	0.003	Heterogeneous nuclear ribonucleoprotein K
P05141	2.807	0.003	ADP/ATP translocase 2
Q6LDX7	13.007	0.003	Tyrosine-protein kinase receptor
Q99623	14.381	0.003	Prohibitin-2
P06733	2.361	0.003	Alpha-enolase
P13639	5.459	0.003	Elongation factor 2
Q15084	43.388	0.003	Protein disulfide-isomerase A6
Q96DV6	3.944	0.003	40S ribosomal protein S6
Q66K53	9.606	0.003	HNRPA3 protein
P15880	4.502	0.003	40S ribosomal protein S2
P39019	5.898	0.004	40S ribosomal protein S19
P63104	2.043	0.004	14-3-3 protein zeta/delta
P22626	6.638	0.004	Heterogeneous nuclear ribonucleoproteins A2/B1
P30101	6.086	0.005	Protein disulfide-isomerase
P25786	8.420	0.005	Proteasome subunit alpha type-1
P11940	12.404	0.006	Polyadenylate-binding protein
P16401	4.877	0.006	Histone H1.5
P07237	5.704	0.006	Protein disulfide-isomerase
Q16777	10.160	0.006	Histone H2A type 2-C;Histone H2A type 2-A
P05386	5.889	0.006	60S acidic ribosomal protein P1
P31948	11.491	0.006	Stress-induced-phosphoprotein 1
P31946	2.156	0.007	14-3-3 protein beta/alpha
P68104	2.558	0.007	Elongation factor 1-alpha
P00338	1.590	0.007	L-lactate dehydrogenase
Q14103	6.189	0.007	Heterogeneous nuclear ribonucleoprotein D0
P38646	10.649	0.007	Stress-70 protein, mitochondrial
P26641	19.766	0.007	Elongation factor 1-gamma
O75347	4.168	0.008	Tubulin-specific chaperone A
P09429	5.878	0.008	High mobility group protein B1
P62942	7.427	0.008	Peptidyl-prolyl cis–trans isomerase FKBP1A
Q9NUV1	7.289	0.008	Cytosolic non-specific dipeptidase
P11021	7.467	0.008	78 kDa glucose-regulated protein
P11142	2.320	0.008	Heat shock cognate 71 kDa protein
P02533	5.320	0.008	Keratin, type I cytoskeletal 14
P30040	6.657	0.008	Endoplasmic reticulum resident protein 29
P50990	11.713	0.008	T-complex protein 1 subunit theta
P46783	9.508	0.008	40S ribosomal protein S10
P31943	14.091	0.008	Heterogeneous nuclear ribonucleoprotein H
P19338	13.679	0.009	Nucleolin
P14625	13.173	0.009	Endoplasmin
Q92597	4.464	0.009	Protein NDRG1
P26599	19.501	0.009	Polypyrimidine tract-binding protein 1
P68363	2.317	0.009	Tubulin alpha-1B chain
P61604	9.723	0.009	10 kDa heat shock protein, mitochondrial
P08238	8.920	0.009	Heat shock protein HSP 90-beta
Q00839	15.338	0.009	Heterogeneous nuclear ribonucleoprotein U
P04843	64.275	0.009	Dolichyl-diphosphooligosaccharide–protein glycosyltransferase subunit 1
P09651	10.489	0.010	Heterogeneous nuclear ribonucleoprotein A1
P22314	3.758	0.010	Ubiquitin-like modifier-activating enzyme 1
P30085	3.180	0.010	UMP-CMP kinase
P23246	39.026	0.011	Splicing factor, proline- and glutamine-rich
P29692	13.726	0.011	Elongation factor 1-delta
P27797	7.508	0.011	Calreticulin
Q06830	1.788	0.011	Peroxiredoxin-1
P84243	2.541	0.012	Histone H3
P05023	15.342	0.012	Sodium/potassium-transporting ATPase subunit alpha-1
Q14974	3.995	0.014	Importin subunit beta-1
P30154	2.882	0.014	Serine/threonine-protein phosphatase 2A
P49448	5.013	0.015	Glutamate dehydrogenase
P20700	14.379	0.015	Lamin-B1
P55072	6.054	0.016	Transitional endoplasmic reticulum ATPase
P35579	8.278	0.016	Myosin-9
P40227	8.241	0.016	T-complex protein 1 subunit zeta
P13010	223.628	0.017	X-ray repair cross-complementing protein 5
Q03252	12.919	0.017	Lamin-B2
P27824	9.105	0.017	Calnexin
P02545	1.376	0.017	Prelamin-A/C;Lamin-A/C
P67936	10.102	0.017	Tropomyosin alpha-4 chain
P04908	2.018	0.018	Histone H2A
P13797	5.684	0.019	Plastin-3
P52907	3.377	0.019	F-actin-capping protein subunit alpha-1
P63241	4.197	0.019	Eukaryotic translation initiation factor 5A
P62491	3.628	0.019	Ras-related protein Rab-11A;Ras-related protein Rab-11B
P45880	2.304	0.020	Voltage-dependent anion-selective channel protein 2
P05387	4.257	0.020	60S acidic ribosomal protein P2
Q5SRT3	3.484	0.021	Chloride intracellular channel protein
P07437	3.687	0.021	Tubulin beta chain
P23284	8.401	0.022	Peptidyl-prolyl cis–trans isomerase
P18124	5.442	0.022	60S ribosomal protein L7
P07355	1.909	0.022	Annexin;Annexin A2
P46777	12.124	0.023	60S ribosomal protein L5
Q99714	1.923	0.023	3-hydroxyacyl-CoA dehydrogenase type-2
O75531	9.745	0.024	Barrier-to-autointegration factor
Q14697	21.165	0.025	Neutral alpha-glucosidase AB
P62263	6.347	0.025	40S ribosomal protein S14
P0DMV9	2.049	0.026	Heat shock 70 kDa protein 1B
P29034	6.458	0.026	Protein S100-A2
P62888	2.893	0.026	60S ribosomal protein L30
Q6IBT3	23.335	0.027	T-complex protein 1 subunit eta
P47756	2.818	0.027	F-actin-capping protein subunit beta
P35222	7.555	0.028	Catenin beta-1
P07339	5.983	0.029	Cathepsin D
Q86SZ7	4.151	0.029	Proteasome activator complex subunit 2
P15311	3.903	0.029	Ezrin;Tyrosine-protein kinase receptor
P59665	4.537	0.029	Neutrophil defensin 1
P09960	5.492	0.030	Leukotriene A-4 hydrolase
P63220	4.048	0.030	40S ribosomal protein S21
Q16658	114.974	0.031	Fascin
P07954	5.399	0.032	Fumarate hydratase, mitochondrial
P54819	4.652	0.034	Adenylate kinase 2, mitochondrial
P07737	1.223	0.034	Profilin-1
P63313	5.261	0.034	Thymosin beta-10
P21796	3.716	0.034	Voltage-dependent anion-selective channel protein 1
P61247	12.449	0.035	40S ribosomal protein S3a
P14618	1.508	0.035	Pyruvate kinase
P61626	4.029	0.036	Lysozyme;Lysozyme C
Q15181	8.459	0.037	Inorganic pyrophosphatase
P27348	3.220	0.037	14-3-3 protein theta
P49411	14.069	0.037	Elongation factor Tu, mitochondrial
P05164	10.019	0.037	Myeloperoxidase
P61160	5.976	0.038	Actin-related protein 2
Q04917	4.768	0.039	14-3-3 protein eta
P62805	1.761	0.039	Histone H4
P26373	3.700	0.040	60S ribosomal protein L13
Q14204	2.799	0.041	Cytoplasmic dynein 1 heavy chain 1
P56537	7.504	0.041	Eukaryotic translation initiation factor 6
P08708	10.144	0.042	40S ribosomal protein S17
P15153	2.613	0.042	Ras-related C3 botulinum toxin substrate 2
P31949	2.100	0.045	Protein S100
P36952	6.679	0.046	Serpin B5
Q15149	4.694	0.047	Plectin
P46779	6.182	0.048	60S ribosomal protein L28
Q59FH0	5.442	0.048	Histone H2A
P62937	1.778	0.049	Peptidyl-prolyl cis–trans isomerase
P07741	5.077	0.049	Adenine phosphoribosyltransferase
P62269	3.688	0.050	40S ribosomal protein S18

**Table 4:** List of 40 proteins that were low-expressed in ESCC tissues

IDs	Log ratio	*P* value	Protein names
P55268	0.078	0.001	Laminin subunit beta-2
Q13361	0.000	0.001	Microfibrillar-associated protein 5
O95682	0.000	0.001	Tenascin-X
P12277	0.024	0.001	Creatine kinase B-type
P20774	0.018	0.002	Mimecan
P06396	0.501	0.002	Gelsolin
O75106	0.000	0.002	Membrane primary amine oxidase
P60660	0.260	0.002	Myosin light polypeptide 6
P51884	0.118	0.003	Lumican
P35555	0.183	0.003	Fibrillin-1
Q5U0D2	0.081	0.004	Transgelin
P35749	0.029	0.004	Myosin-11
P51888	0.032	0.004	Prolargin
P24844	0.033	0.005	Myosin regulatory light polypeptide 9
P17661	0.063	0.005	Desmin
P98160	0.213	0.006	Basement membrane-specific heparan sulfate proteoglycan core protein
P12109	0.299	0.006	Collagen alpha-1(VI) chain
Q07507	0.084	0.006	Dermatopontin
P11047	0.209	0.006	Laminin subunit gamma-1
Q6ZN40	0.114	0.006	CDNA FLJ16459 fis
P18206	0.259	0.008	Vinculin
Q14112	0.065	0.010	Nidogen-2
P21291	0.086	0.011	Cysteine and glycine-rich protein 1
P68032	0.312	0.011	Actin, alpha cardiac muscle 1
Q9NZN4	0.000	0.012	EH domain-containing protein 2
P07585	0.087	0.012	Decorin
Q15746	0.021	0.014	Myosin light chain kinase, smooth muscle
Q9Y490	0.318	0.015	Talin-1
P12110	0.223	0.016	Collagen alpha-2(VI) chain
P21810	0.235	0.020	Biglycan
Q93052	0.048	0.021	Lipoma-preferred partner
P30086	0.507	0.021	Phosphatidylethanolamine-binding protein 1
P62736	0.043	0.022	Actin, aortic smooth muscle
Q96AC1	0.029	0.023	Fermitin family homolog 2
Q6NZI2	0.213	0.025	Polymerase I and transcript release factor
Q59F18	0.000	0.027	Smoothelin isoform b variant
O14558	0.000	0.027	Heat shock protein beta-6
Q13642	0.004	0.028	Four and a half LIM domains protein 1
P12111	0.321	0.031	Collagen alpha-3(VI) chain
P29536	0.000	0.032	Leiomodin-1
P05556	0.416	0.033	Integrin beta-1
Q15124	0.000	0.033	Phosphoglucomutase-like protein 5
P21333	0.213	0.033	Filamin-A
Q53GG5	0.013	0.036	PDZ and LIM domain protein 3
P01009	0.429	0.037	Alpha-1-antitrypsin;Short peptide from AAT
P43121	0.000	0.038	Cell surface glycoprotein MUC18
P52943	0.210	0.041	Cysteine-rich protein 2
P08294	0.000	0.043	Extracellular superoxide dismutase [Cu–Zn]
P56539	0.155	0.043	Caveolin
O15061	0.000	0.045	Synemin
Q9NR12	0.044	0.047	PDZ and LIM domain protein 7

### Bioinformatics analysis of differentially expressed proteins

**Fig. 2:**
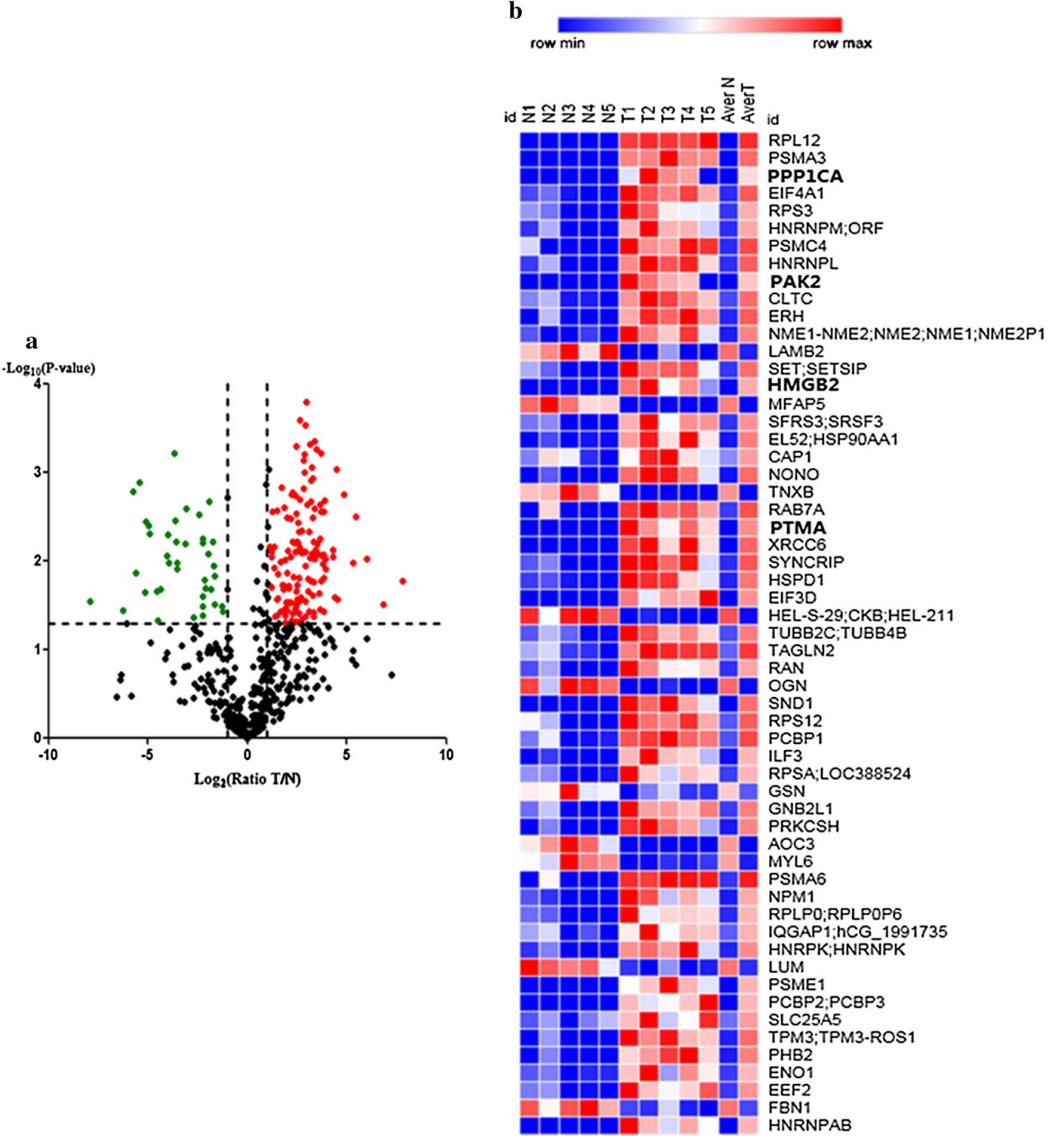
Analysis of protein differential expression. **a** Volcano plot graph illustrating the differential abundant proteins in the quantitative analysis. The − log10 (*P* value) was plotted against the log2 (ratio cancer/normal). The red dots represented proteins up-regulated in cancer samples, green dots corresponded to proteins down-regulated in cancer samples. **b** The heat map of significantly different proteins was shown between cancer tissues and adjacent normal tissues. The analysis was achieved by using Morpheus (https://software.broadinstitute.org/morpheus)

**Fig. 3:**
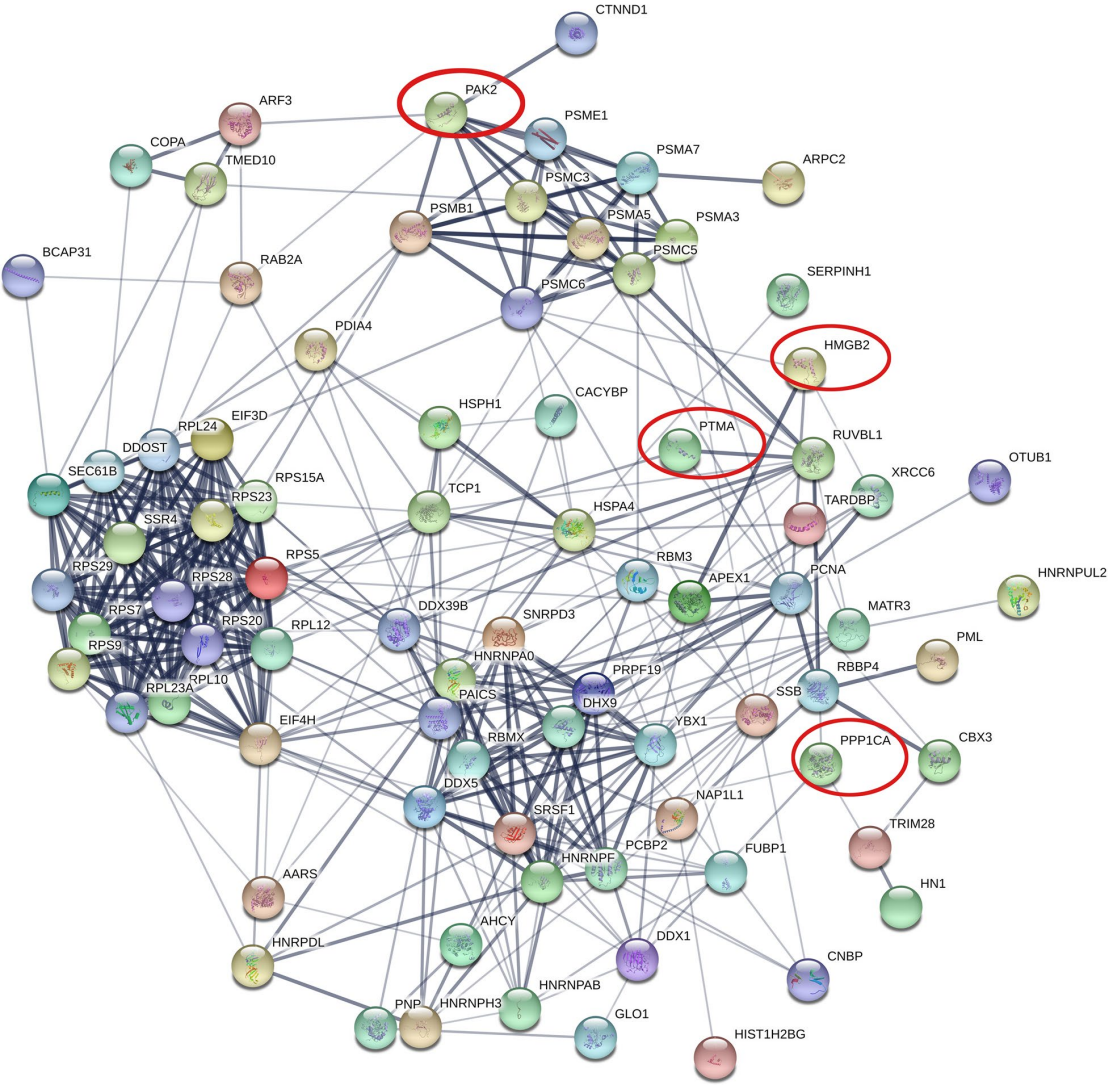
Protein-protein interaction network of the differently expressed proteins was identified by STRING. Four proteins were selected for further study with filled red circles (https://string-db.org/)

**Fig. 4:**
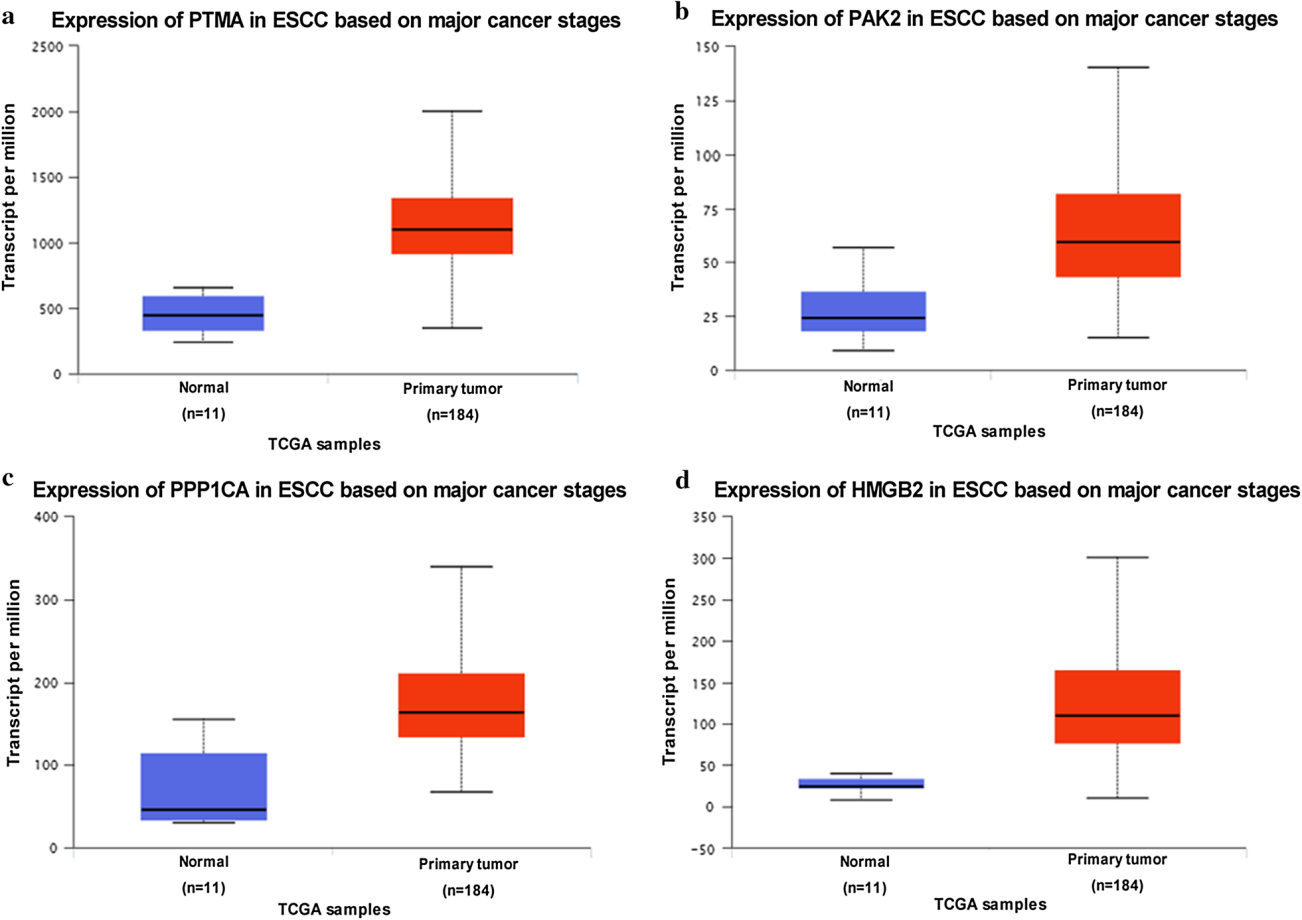
The expression of PTMA, PAK2, PPP1CA and HMGB2 in ESCC based on major cancer stages. In the TCGA databases, the four genes were up-regulated in EC patients (*P *< 0.001). (http://ualcan.path.uab.edu/analysis.html)

A volcano plot was generated based on the differential expression ratio and *P* value (Fig. 2a). Moreover, the heat map of significantly different proteins was shown in Fig. 2b by using Morpheus (https://software.broadinstitute.org/morpheus). Further protein–protein interaction analysis of the differently expressed proteins was performed by STRING, the result was shown in Fig. 3. Out of the four proteins selected for next analysis, the PPI network analysis revealed that PTMA was a valid target of c-myc transcriptional activation, while PPP1CA was involved in down-regulation of TGF-beta receptor signaling. PAK2 plays a role in apoptosis and activation of Rac, while HMGB2 is participating in chromatin regulation and retinoblastoma in cancer. Above mentioned, all these four proteins were associated with the occurrence and development of cancer. Bioinformatics analysis of the four genes from TCGA database revealed that the four genes up-regulated in gene level in EC tissue (Fig. 4). Whether these four genes can be used as biomarkers of esophageal cancer remains to be further studied.

### Validation of differentially expressed proteins by Western Blot

**Fig. 5:**
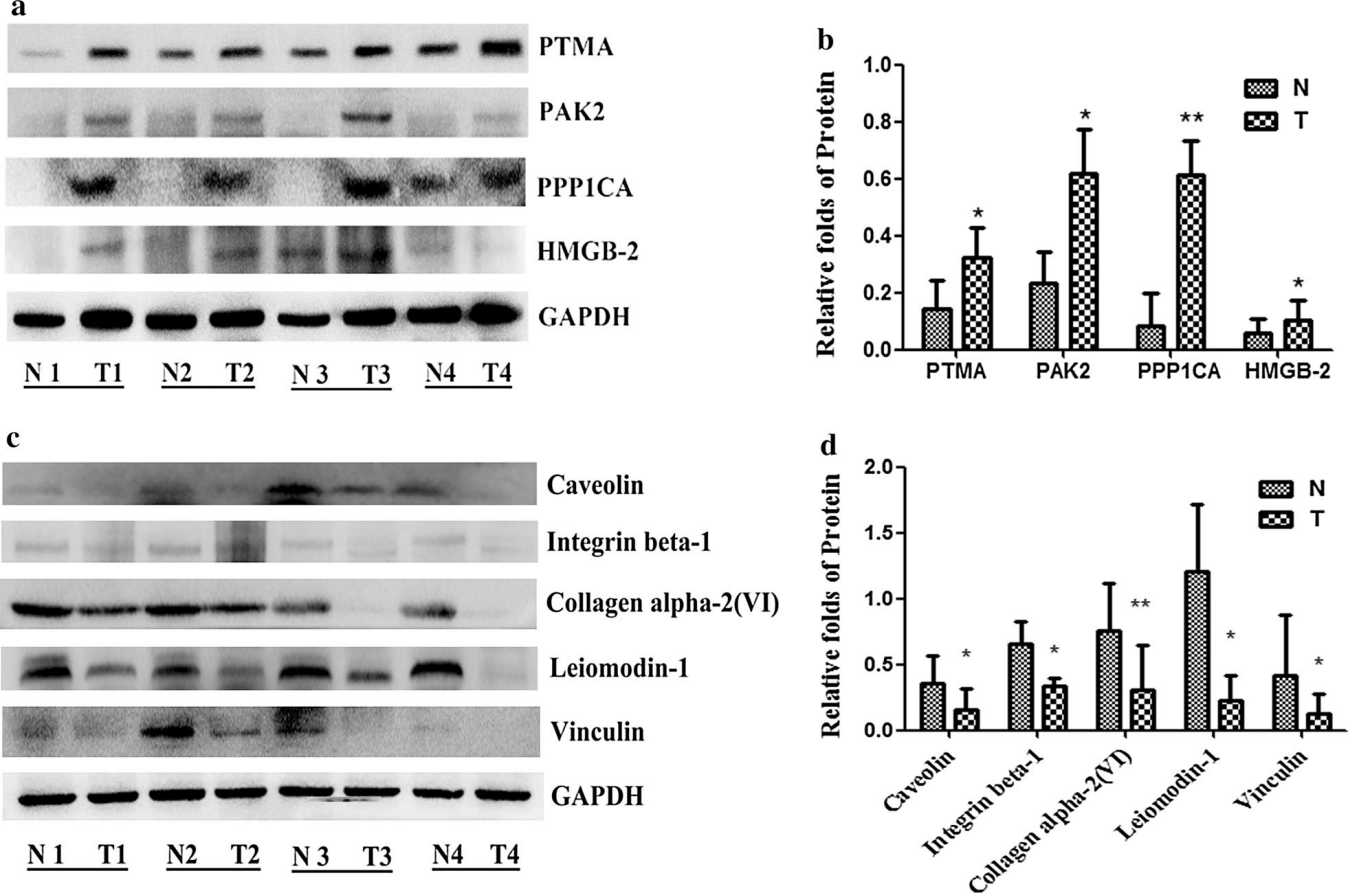
The differentially expressed proteins were validated by Western Blot. Compared with adjacent normal tissues, the protein expression of PTMA, PAK2, PPP1CA, HMGB2 were up-regulated (**a**, **b**), and the protein expression of Caveolin, Integrin beta-1, Collagen alpha-2(VI), Leiomodin-1, Vinculin were down-regulated in ESCC tissues from four pairs of samples (**c**, **d**). Representative immunoblot images (**a**, **c**) and histograms (mean ± SD; **b**, **d**).The experiments were repeated at least three times, N represented normal tissues and T represented tumor tissues

To further validate the LC–MS/MS results, we evaluated the four up-regulated proteins (PTMA, PAK2, PPP1CA, HMGB2) and the five down-regulated proteins [Caveolin, Integrin beta-1, Collagen alpha-2(VI), Leiomodin-1 and Vinculin] with Western Blot on the same samples. Compared with adjacent normal tissues, the protein expression of PTMA, PAK2, PPP1CA, HMGB2 were up-regulated (Fig. 5a, b), and the protein expression of Caveolin, Integrin beta-1, Collagen alpha-2(VI), Leiomodin-1, Vinculin were down-regulated in ESCC tissues from four pairs of samples (Fig. 5c, d). The results showed that the trends expression of these proteins were consistent with the LC–MS results.

### Validation of PTMA involved in ESCC by QDB and IHC

**Fig. 6:**
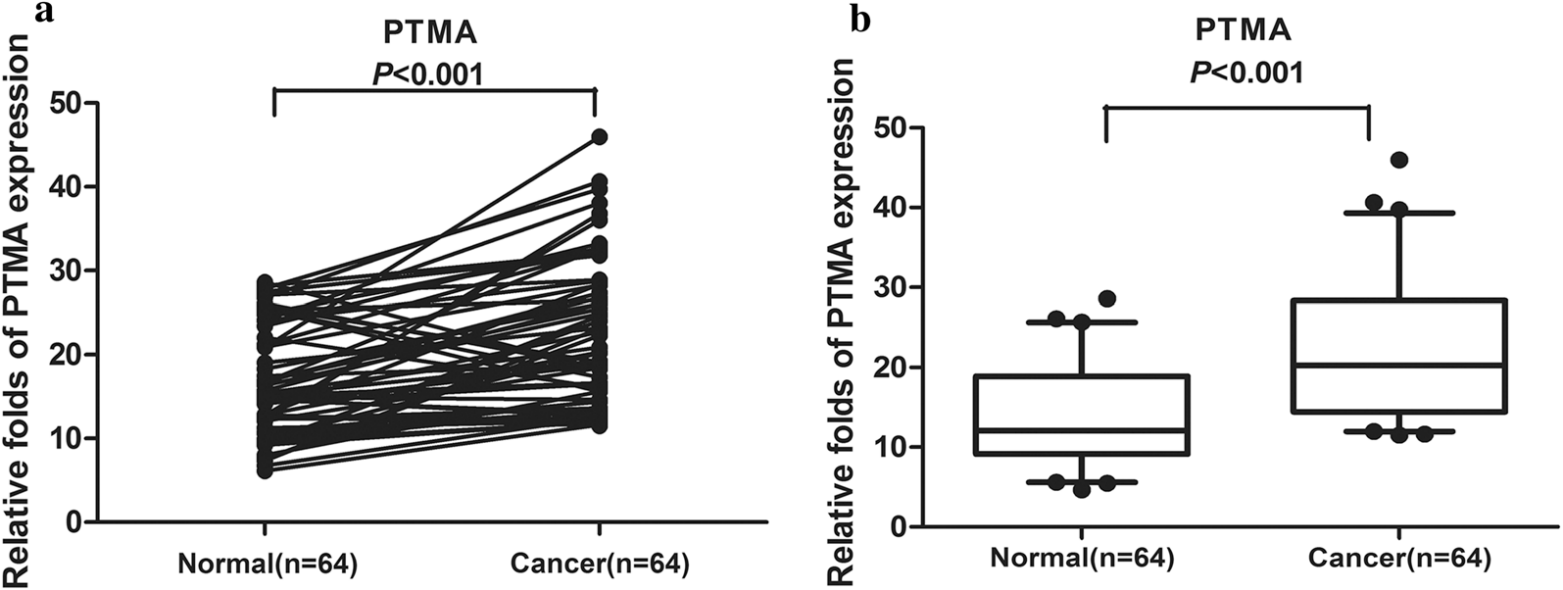
The relative PTMA expression was tested by QDB in ESCC and adjacent normal tissues from 64 esophageal cancer patients. **a** The differential expression of PTMA was shown in each pair of tissues. **b** The PTMA expression was up-regulated in esophageal cancer tissues from the average of 64 pairs of tissues

**Fig. 7:**
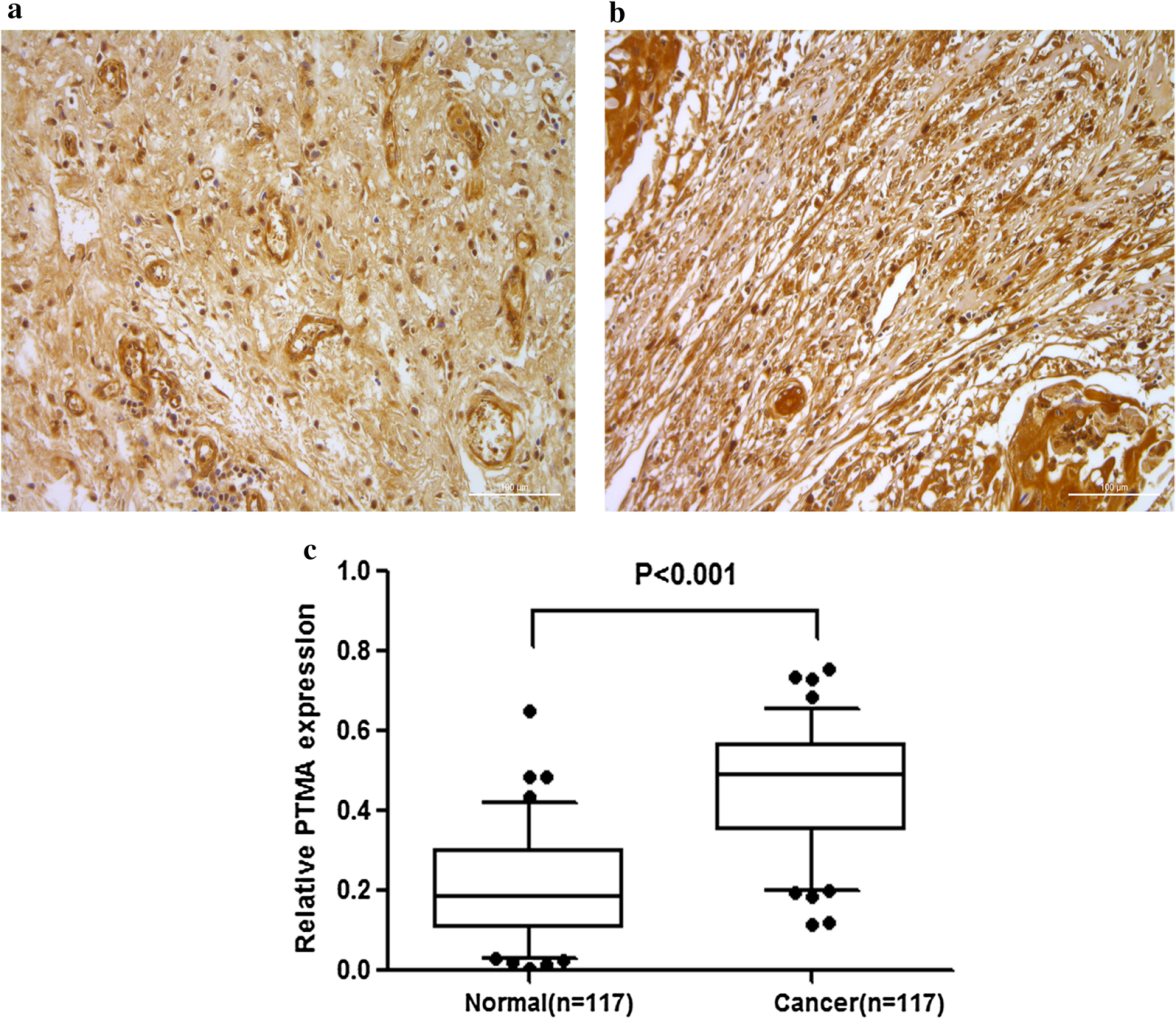
The relative PTMA expression was tested by IHC in ESCC and adjacent normal tissues among 117 pairs of tissues (× 200). **a** The expression of PTMA in adjacent normal tissues were presented. **b** The expression of PTMA in esophageal cancer were up-regulated. **c** The gray-scale analysis of immunohistochemical results (*P *< 0.001)

**Fig. 8:**
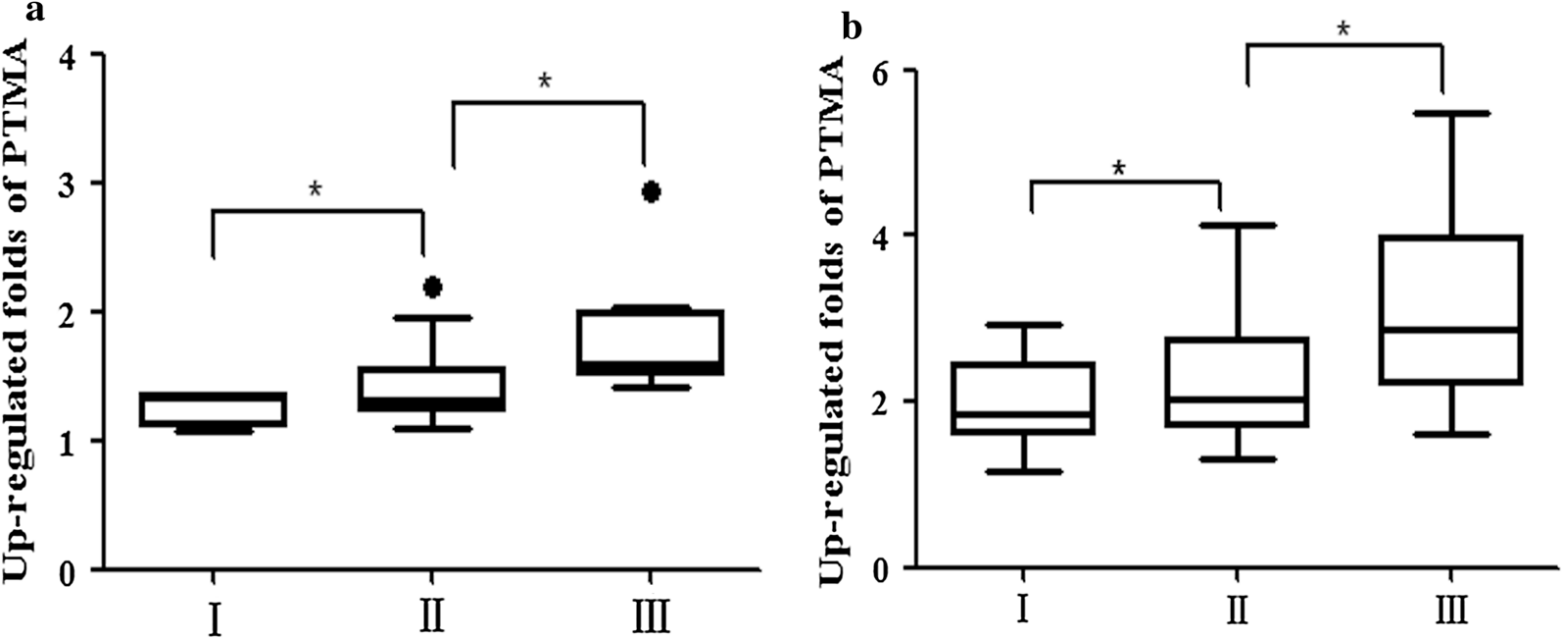
The PTMA expression was up-regulated gradually along the progression of ESCC. **a** The PTMA expression trend at the different Grades in QDB samples. **b** The PTMA expression trend at the different Grades in IHC samples. I, II, III represented ESCC Grade I, Grade II and Grade III respectively. (*P < 0.05)

In order to validate the proteins identified by mass spectrometric, the QDB technique was applied in a larger set of samples. We collected the samples of 64 patients, and the relevant clinical information was summarized in Table 5. In the analysis of 64 patient samples, we found that 53 out of 64 esophageal cancer tissues showed higher PTMA expression than in the normal tissues (*P *< 0.001) (Fig. 6). This trend was in accordance with the previous data. To further validate the QDB results, we performed the tissue microarray analysis by IHC. The results showed that among 117 pairs of tissues, the high expression rate of PTMA in tumor tissues was 98% (115/117). A significant overexpression of PTMA was found in tumor tissues in contrast to adjacent normal tissues (*P *< 0.01) (Fig. 7). The sample information in the chip is summarized in Tables 6 and 7. We further evaluated the expression pattern of PTMA with the progression, and analyzed the PTMA expression trend in the different tumor Grades. The results revealed that the PTMA expression was up-regulated gradually along the progression of ESCC (Fig. 8). The PTMA expression ratio between tumor and adjacent normal tissue was significantly increased along with the progression (*P *< 0.05). So we can suspect that PTMA might be participating in the development of esophageal cancer.

**Table 5:** The clinical features of ESCC patients for QDB analysis

No.	Gender	Age	Organ/anatomic site	Grade	TNM
1	Male	69	esophagus	II	T1N0M0
2	Male	61	esophagus	I	T0N0M0
3	Male	59	esophagus	II	T3N0M0
4	Female	65	esophagus	I	T0N0M0
5	Male	52	esophagus	II–III	T3N0M0
6	Female	73	esophagus	I–II	T1N0M0
7	Male	46	esophagus	I	T0N0M0
8	Male	64	Lower segment of esophagus	II	T3N2M0
9	Male	57	Mid-thoracic esophagus	II	T3N0M0
10	Male	54	Mid-thoracic esophagus	II–III	T3N0M0
11	Male	72	Mid-thoracic esophagus	II	T3N3M0
12	Male	66	Mid-thoracic esophagus	II	T3N3M0
13	Male	62	Middle-lower esophagus	II	T1N0M0
14	Male	60	esophagus	II	T3N0M0
15	Female	60	esophagus	II	T3N0M0
16	Male	64	esophagus	II	T3N0M0
17	Female	58	Lower thoracic esophagus	III	T3N0M0
18	Male	53	esophagus	II	T3N0M0
19	Male	65	Lower thoracic esophagus	II–III	T3N0M0
20	Female	60	Mid-thoracic esophagus	I–III	T3N0M0
21	Male	69	Middle-lower esophagus	II	T3N3M0
22	Female	66	esophagus	II–III	T3N2M0
23	Female	67	Lower segment of esophagus	II–III	T3N3M1
24	Male	67	Mid-thoracic esophagus	III	T3N1M0
25	Female	55	Mid-thoracic esophagus	II	T2N1M0
26	Female	61	Mid-thoracic esophagus	I–II	T1N2M0
27	Male	68	esophagus	II–III	T3N2M0
28	Female	48	Mid-thoracic esophagus	I–II	T3N0M0
29	Female	63	Mid-thoracic esophagus	II	T1N1M0
30	Male	70	Lower segment of esophagus	II	T2N1M0
31	Female	59	Mid-thoracic esophagus	III	T3N1M0
32	Female	48	Mid-thoracic esophagus	II	T3N0M0
33	Female	53	Mid-thoracic esophagus	II	T3N2M1
34	Female	58	Lower thoracic esophagus	I-II	T3N0M0
35	Male	62	Mid-thoracic esophagus	II	T2N0M0
36	Female	59	esophagus	II	T3N1M1
37	Female	57	esophagus	II	T3N0M0
38	Female	57	Lower thoracic esophagus	II	T3N1M1
39	Female	62	Mid-thoracic esophagus	I–II	T3N0M0
40	Female	69	Mid-thoracic esophagus	II–III	T3N1M1
41	Female	61	Mid-thoracic esophagus	II	T3N2M1
42	Female	67	Mid-thoracic esophagus	II	T2N0M0
43	Female	47	Mid-thoracic esophagus	II	T2N0M0
44	Female	69	Lower thoracic esophagus	III	T2N2M1
45	Male	66	esophagus	II	T3N0M0
46	Male	72	Mid-thoracic esophagus	II	T3N0M0
47	Female	69	Mid-thoracic esophagus	II–III	T3N0M0
48	Female	73	Mid-thoracic esophagus	I	T1N0M0
49	Male	62	esophagus	II	T3N0M0
50	Male	58	esophagus	II	T2N0M0
51	Male	56	Lower segment of esophagus	II	T1N0M0
52	Male	56	Middle-lower esophagus	II	T3N0M0
53	Male	56	Middle-lower esophagus	II	T3N0M0
54	Male	55	esophagus	I–II	T3N0M0
55	Female	61	esophagus	I–II	T3N0M0
56	Female	71	Middle-lower esophagus	I–II	T1N0M0
57	Male	61	esophagus	II–III	T3N3M1
58	Male	62	Upper thoracic esophagus	III	T3N0M0
59	Male	67	Mid-thoracic esophagus	I	T1N0M0
60	Male	65	esophagus	I	T3N0M0
61	Male	58	esophagus	II–III	T2N1M1
62	Male	49	Lower segment of esophagus	I	T1N0M0
63	Female	66	esophagus	III	T3N1M1
64	Male	70	esophagus	I	T1N0M0

**Table 6:** The 35 pairs samples in tissue microarrays (TMA) (ES701) for immunohistochemistry analysis

No.	Gender	Age	Organ/anatomic site	Grade	TNM
1	Male	60	Esophagus	II	T3N1M0
2	Male	60	Esophagus	–	–
3	Male	44	Esophagus	I	T3N1M0
4	Male	44	Esophagus	–	–
5	Male	50	Esophagus	I	T3N2M0
6	Male	50	Esophagus	–	–
7	Male	53	Esophagus	I	T3N0M0
8	Male	53	Esophagus	–	–
9	Male	64	Esophagus	I	T3N1M0
10	Male	64	Esophagus	–	–
11	Male	69	Esophagus	I	T3N0M0
12	Male	69	Esophagus	–	–
13	Male	59	Esophagus	I	T3N0M0
14	Male	59	Esophagus	–	–
15	Male	60	Esophagus	I	T3N1M0
16	Male	60	Esophagus	–	–
17	Male	72	Esophagus	I	T3N1M0
18	Male	72	Esophagus	–	–
19	Female	60	Esophagus	I	T3N1M0
20	Female	60	Esophagus	–	–
21	Female	75	Esophagus	III	T3N0M0
22	Female	75	Esophagus	–	–
23	Male	57	Esophagus	II	T3N1M0
24	Male	57	Esophagus	–	–
25	Female	54	Esophagus	II	T3N1M0
26	Female	54	Esophagus	–	–
27	Male	45	Esophagus	III	T3N0M0
28	Male	45	Esophagus	–	–
29	Male	52	Esophagus	II	T3N0M0
30	Male	52	Esophagus	–	–
31	Male	68	Esophagus	–	T3N0M0
32	Male	68	Esophagus	–	–
33	Male	67	Esophagus	I	T3N0M0
34	Male	67	Esophagus	–	–
35	Male	55	Esophagus	I	T3N0M0
36	Male	55	Esophagus	–	–
37	Male	71	Esophagus	I	T3N1M0
38	Male	71	Esophagus	–	–
39	Male	63	Esophagus	III	T3N1M0
40	Male	63	Esophagus	–	–
41	Male	67	Esophagus	III	T3N1M0
42	Male	67	Esophagus	–	–
43	Male	57	Esophagus	III	T3N0M0
44	Male	57	Esophagus	–	–
45	Male	63	Esophagus	III	T3N0M0
46	Male	63	Esophagus	–	–
47	Male	57	Esophagus	III	T3N1M0
48	Male	57	Esophagus	–	–
49	Male	58	Esophagus	III	T3N1M0
50	Male	58	Esophagus	–	–
51	Male	53	Esophagus	II	T3N1M0
52	Male	53	Esophagus	–	–
53	Male	49	Esophagus	I	T3N1M0
54	Male	49	Esophagus	–	–
55	Male	68	Esophagus	III	T3N1M0
56	Male	68	Esophagus	–	–
57	Male	48	Esophagus	III	T3N0M0
58	Male	48	Esophagus	–	–
59	Female	58	Esophagus	II	T3N1M0
60	Female	58	Esophagus	–	–
61	Male	44	Esophagus	III	T3N1M0
62	Male	44	Esophagus	–	–
63	Male	63	Esophagus	II	T3N1M0
64	Male	63	Esophagus	–	–
65	Male	68	Esophagus	III	T3N1M0
66	Male	68	Esophagus	–	–
67	Female	68	Esophagus	III	T3N1M0
68	Female	68	Esophagus	–	–
69	Male	62	Esophagus	III	T2M1N1B
70	Male	62	Esophagus	–	–

**Table 7:** The 96 pairs samples in tissue microarrays (TMA) (ES1922) for immunohistochemistry analysis

No.	Gender	Age	Organ/anatomic site	Grade	TNM
1	Male	58	Esophagus	I	T3N0M0
2	Male	58	Esophagus	–	–
3	Male	68	Esophagus	I	T3N1M0
4	Male	68	Esophagus	–	–
5	Male	52	Esophagus	I	T1N0M0
6	Male	52	Esophagus	–	–
7	Female	66	Esophagus	I	T3N0M0
8	Female	66	Esophagus	–	–
9	Male	72	Esophagus	I	T3N1M0
10	Male	72	Esophagus	–	–
11	Male	67	Esophagus	I	T3N0M0
12	Male	67	Esophagus	–	–
13	Male	66	Esophagus	I	T3N1M0
14	Male	66	Esophagus	–	–
15	Male	55	Esophagus	I	T3N1M0
16	Male	55	Esophagus	–	–
17	Male	67	Esophagus	I	T3N1M0
18	Male	67	Esophagus	–	–
19	Female	71	Esophagus	I	T3N0M0
20	Female	71	Esophagus	–	–
21	Male	69	Esophagus	I	T3N0M0
22	Male	69	Esophagus	–	–
23	Male	68	Esophagus	I	T3N0M0
24	Male	68	Esophagus	–	–
25	Male	44	Esophagus	I	T3N1M0
26	Male	44	Esophagus	–	–
27	Female	63	Esophagus	I	T2N0M0
28	Female	63	Esophagus	–	–
29	Female	54	Esophagus	I	T3N1M0
30	Female	54	Esophagus	–	–
31	Male	60	Esophagus	I	T2N0M0
32	Male	60	Esophagus	–	–
33	Female	68	Esophagus	II	T3N0M0
34	Female	68	Esophagus	–	–
35	Male	49	Esophagus	I	T3N1M0
36	Male	49	Esophagus	–	–
37	Male	61	Esophagus	I	T3N0M0
38	Male	61	Esophagus	–	–
39	Female	69	Esophagus	I	T3N1M0
40	Female	69	Esophagus	–	–
41	Male	49	Esophagus	I	T3N1M0
42	Male	49	Esophagus	–	–
43	Male	68	Esophagus	I	T3N0M0
44	Male	68	Esophagus	–	–
45	Male	66	Esophagus	II	T3N0M0
46	Male	66	Esophagus	–	–
47	Male	53	Esophagus	II	T3N1M0
48	Male	53	Esophagus	–	–
49	Female	58	Esophagus	I	T3N0M0
50	Female	58	Esophagus	–	–
51	Male	63	Esophagus	I	T3N0M0
52	Male	63	Esophagus	–	–
53	Female	68	Esophagus	I	T2N0M0
54	Female	68	Esophagus	–	–
55	Female	68	Esophagus	I	T3N0M0
56	Female	68	Esophagus	–	–
57	Male	58	Esophagus	I	T3N0M0
58	Male	58	Esophagus	–	–
59	Female	60	Esophagus	I	T3N0M0
60	Female	60	Esophagus	–	–
61	Male	70	Esophagus	II	T2N1M0
62	Male	70	Esophagus	–	–
63	Female	61	Esophagus	I	T3N0M0
64	Female	61	Esophagus	–	–
65	Male	54	Esophagus	II	T3N0M0
66	Male	54	Esophagus	–	–
67	Male	45	Esophagus	II	T3N0M0
68	Male	45	Esophagus	–	–
69	Male	75	Esophagus	III	T3N0M0
70	Male	75	Esophagus	–	–
71	Male	63	Esophagus	I	T3N0M0
72	Male	63	Esophagus	–	–
73	Male	68	Esophagus	I	T3N0M0
74	Male	68	Esophagus	–	–
75	Female	50	Esophagus	II	T3N0M0
76	Female	50	Esophagus	–	–
77	Male	72	Esophagus	III	T3N0M0
78	Male	72	Esophagus	–	–
79	Female	53	Esophagus	III	T3N0M0
80	Female	53	Esophagus	–	–
81	Male	69	Esophagus	II	T3N1M0
82	Male	69	Esophagus	–	–
83	Male	57	Esophagus	I	T3N0M0
84	Male	57	Esophagus	–	–
85	Male	68	Esophagus	III	T3N1M0
86	Male	68	Esophagus	–	–
87	Male	51	Esophagus	III	T3N0M0
88	Male	51	Esophagus	–	–
89	Male	70	Esophagus	I	T3N1M0
90	Male	70	Esophagus	–	–
91	Male	68	Esophagus	II	T3N1M0
92	Male	68	Esophagus	–	–
93	Male	57	Esophagus	III	T3N0M0
94	Male	57	Esophagus	–	–
95	Male	48	Esophagus	II	T3N0M0
96	Male	48	Esophagus	–	–
97	Male	63	Esophagus	III	T3N1M0
98	Male	63	Esophagus	–	–
99	Male	65	Esophagus	II	T3N0M0
100	Male	65	Esophagus	–	–
101	Male	71	Esophagus	III	T3N1M0
102	Male	71	Esophagus	–	–
103	Male	78	Esophagus	III	T3N0M0
104	Male	78	Esophagus	–	–
105	Male	53	Esophagus	II	T3N1M0
106	Male	53	Esophagus	–	–
107	Male	57	Esophagus	II	T3N0M0
108	Male	57	Esophagus	–	–
109	Male	63	Esophagus	II	T3N1M0
110	Male	63	Esophagus	–	–
111	Male	63	Esophagus	III	T3N1M0
112	Male	63	Esophagus	–	–
113	Female	58	Esophagus	I	T3N1M0
114	Female	58	Esophagus	–	–
115	Male	50	Esophagus	II	T2N0M0
116	Male	50	Esophagus	–	–
117	Male	44	Esophagus	I	T3N1M0
118	Male	44	Esophagus	–	–
119	Male	61	Esophagus	I	T3N1M0
120	Male	61	Esophagus	–	–
121	Male	61	Esophagus	I	T3N1M0
122	Male	61	Esophagus	–	–
123	Male	57	Esophagus	II	T3N1M0
124	Male	57	Esophagus	–	–
125	Male	60	Esophagus	I	T3N0M0
126	Male	60	Esophagus	–	–
127	Male	58	Esophagus	II	T3N0M0
128	Male	58	Esophagus	–	–
129	Male	61	Esophagus	II	T3N0M0
130	Male	61	Esophagus	–	–
131	Male	52	Esophagus	I	T3N1M0
132	Male	52	Esophagus	–	–
133	Female	60	Esophagus	II	T3N1M0
134	Female	60	Esophagus	–	–
135	Male	68	Esophagus	II	T3N0M0
136	Male	68	Esophagus	–	–
137	Female	43	Esophagus	III	T3N1M0
138	Female	43	Esophagus	–	–
139	Male	59	Esophagus	III	T3N1M0
140	Male	59	Esophagus	–	–
141	Male	55	Esophagus	III	T3N1M0
142	Male	55	Esophagus	–	–
143	Male	68	Esophagus	III	T3N0M0
144	Male	68	Esophagus	–	–
145	Female	70	Esophagus	III	T3N0M0
146	Female	70	Esophagus	–	–
147	Male	74	Esophagus	III	T2N0M0
148	Male	74	Esophagus	–	–
149	Male	54	Esophagus	I	T2N0M0
150	Male	54	Esophagus	–	–
151	Male	64	Esophagus	III	T3N1M0
152	Male	64	Esophagus	–	–
153	Male	57	Esophagus	I	T3N1M0
154	Male	57	Esophagus	–	–
155	Male	48	Esophagus	III	T3N0M0
156	Male	48	Esophagus	–	–
157	Female	61	Esophagus	III	T3N0M0
158	Female	61	Esophagus	–	–
159	Male	61	Esophagus	III	T3N1M0
160	Male	61	Esophagus	–	–
161	Male	65	Esophagus	III	T3N0M0
162	Male	65	Esophagus	–	–
163	Male	55	Esophagus	III	T2N0M0
164	Male	55	Esophagus	–	–
165	Female	56	Esophagus	III	T3N0M0
166	Female	56	Esophagus	–	–
167	Female	73	Esophagus	II	T3N0M0
168	Female	73	Esophagus	–	–
169	Male	70	Esophagus	III	T3N0M0
170	Male	70	Esophagus	–	–
171	Male	53	Esophagus	III	T3N1M0
172	Male	53	Esophagus	–	–
173	Male	67	Esophagus	III	T2N0M0
174	Male	67	Esophagus	–	–
175	Male	69	Esophagus	III	T3N0M0
176	Male	69	Esophagus	–	–
177	Male	68	Esophagus	III	T3N0M0
178	Male	68	Esophagus	–	–
179	Male	64	Esophagus	III	T3N0M0
180	Male	64	Esophagus	–	–
181	Male	61	Esophagus	III	T3N1M0
182	Male	61	Esophagus	–	–
183	Male	59	Esophagus	III	T3N0M0
184	Male	59	Esophagus	–	–
185	Male	57	Esophagus	III	T2N0M0
186	Male	57	Esophagus	–	–
187	Male	64	Esophagus	III	T3N0M0
188	Male	64	Esophagus	–	–
189	Female	67	Esophagus	I	T2N0M0
190	Female	67	Esophagus	–	–
191	Male	47	Esophagus	III	T2N0M0
192	Male	47	Esophagus	–	–

## Discussions

At present, most patients with esophageal cancer are diagnosed at the late and advanced stages [17]. It is thus urgent to reveal biomarkers related to the progression of esophageal cancer for early diagnosis. Recently, several biomarkers were identified in EC detection, diagnosis, treatment and prognosis. For example, the epidermal growth factor receptor (EGFR), vascular endothelial growth factor (VEGF) and estrogen receptor (ER) were important detection factors for immunohistochemistry in EC [18–20]. In blood, the serum p53 antibody had a potential diagnostic value for EC, however, the detection was limited by its low sensitivity [21]. Therefore, we need to discover and verify more biomarker candidates for the prediction, diagnosis, treatment and prognosis of esophageal cancer.

Mass spectrometry is an effective method for finding distinct molecular regulators, between normal tissues and cancer tissues [22]. In current study, we proposed a significant proteomics profiling difference including 308 proteins. However, compare to previous tissue-based ESCC proteomics study, a poor overlap of proteome profiling was noticed. There are several potential reasons. First, like many other cancers, ESCC is a heterogeneous cancer with different gene expression profiles from different populations [23]. Recently, the whole-genome sequencing revealed the diverse models of structural variations in ESCC, which indicted the biological differences among patients [24]. Therefore, the proteome variation may be a consequence of distinct molecular signatures that exist in ESCC. Another reasons could be related to the different experiment design, some of studies pooled several individual samples into a sample pooling, which would also lead to potential difference compare to our individual analysis [25]. The difference of data analysis method would be another reason too, most of the labeled-based MS approach selected the expression fold change as the major criteria. In our study, with a label-free approach, we proposed paired Student’s *t*-test significance as the main criteria. Such difference could lead to a different proteome profiling. The poor overlap indicated the importance of large-scale validation of biomarker. Thus we suggest in future studies, the proposed novel biomarker should be validated in a larger population no less than 100 samples. Besides TMA, our group recently developed QDB as a novel fast and accurate validation approach, which can easily validate biomarkers up to thousand samples [16].

Human prothymosin-α (PTMA) is a 109 amino acid protein belonged to the α-thymosin family, which is ubiquitously distributed in mammalian blood, tissues and especially abundant in lymphoid cells. However, its role still remains elusive. The growing evidences suggested that PTMA being an important immune mediator as well as a biomarker might eventually become a new therapeutic target or diagnostic method in several diseases such as cancer and inflammation [26]. So we focused on the possibility of PTMA as a biomarker of ESCC.

The proteomic studies show that PTMA exerts multifunction in nuclear and cytoplasmic. In proliferating cells, PTMA mainly locates in nuclear depending on the C-terminus signal sequence, but this protein can be transferred from the nucleus into the cytoplasmic during the cell extraction process [27, 28]. PTMA may mediate the chromatin activity by participated the nuclear-protein complex. In cytoplasmic, the function of PTMA is related to the state of phosphorylation, for example, the Thr7 is the only residue phosphorylated in carcinogenic lymphocytes while the Thr12 or Thr13 phosphorylated in normal lymphocytes [29, 30]. The co-immunoprecipitation experiments shows that PTMA interact with SET, ANP32A and ANP32B to form the complex, which is related to the cell proliferation, membrane trafficking, proteolytic processing and so on [31–33].

PTMA is known to play an important role in cell growth, proliferation, apoptosis and so on [34, 35]. Recent studies have confirmed that overexpression of PTMA is involved in the development of various malignancies, including colorectal, bladder, lung, and liver cancer [36–38]. In vivo tumorigenesis, the PTMA expression promotes the transplant tumor growth in mice and speeds up their death. Meanwhile, the PTMA interacts with TRIM21 directly to regulate the Nrf2 expression through p62/Keap1 signaling in human bladder cancer [39]. In the patients with squamous cell carcinoma (SCC), adenosquamous cell carcinoma (ASC) and adenocarcinoma (AC) of the gallbladder, the positive expression of PTMA may be associated with the tumorigenesis, tumor progression and prognosis in gallbladder tumor. In addition, the high expression of PTMA may be as an indicator in the prevention and early diagnosis of gallbladder tumor [40]. In addition to inducing cancer, Wang et al. discovered that PTMA as a new autoantigen regulated oral submucous fibroblast proliferation and extracellular matrix using human proteome microarray analysis. In addition, PTMA knockdown reversed TGFβ1-induced fibrosis process through reducing the protein levels of collagen I, α-SMA and MMP [34]. However, there have been no evidences that PTMA participates in the pathogenesis of esophageal cancer.

Our mass spectrometry results showed that PTMA expression was up-regulated in ESCC tissues, and if the result was universal, it would provide a good biomarker for the diagnosis of ESCC. The traditional Western Blot is tedious, laborious and time-consuming for hundreds and thousands of large samples tests. In order to verify the results of mass spectrometry, we adopted the QDB technology invented recently, which was capable of high-throughput identification of target proteins from the perspective of biological experiments compared with Western Blot. QDB performed an affordable method for high-throughput immunoblot analysis and achieved relative or absolute quantification. In addition, the QDB needs less sample consumption, and the data can be conveniently read by a microplate reader. In HEK293 cells, the QDB successfully compared the levels of relative p65 levels between Luciferase and p65 clones in 71 pairs of samples. We have confirmed the accuracy and reliability of QDB from both cells and tissues [16]. As above mentioned, QDB is a convenient, reliable and affordable method. In our study, we confirmed that 53 out of 64 tested ESCC tissues had higher PTMA expression by the QDB, and the results were identified by classical IHC methods in 117 pairs of samples.

In this study, we included both explore experiment and validation experiment, using early and late stage samples. The results from explore experiment indicated that PTMA was overexpressed in all stages. We further evaluated the expression pattern of PTMA with the progression, and analyzed the PTMA expression trend in the different Grades. The results revealed that the PTMA expression was up-regulated gradually along the progression of ESCC, and the PTMA expression ratio between tumor and adjacent normal tissue was significantly increased along with the progression. As it is almost impossible to obtain the extreme early stage (such as the stage without any symptom, or the stage prior to Grade I), but from the trend between Grade I and III, we can suspect the expression ratio of PTMA would be a potential indicator for the progression, even in the early diagnosis.

## Conclusions

In our research, we used label-free quantitative proteomics to detect differentially expressed protein profiles in ESCC tissues compared to control tissues. In total 2297 proteins were identified and 308 proteins with significant differences were selected for study. Based on in-depth bioinformatic analysis, the four up-regulated proteins [PTMA, PAK2, PPP1CA, HMGB2) and the five down-regulated proteins Caveolin, Integrin beta-1, Collagen alpha-2(VI), Leiomodin-1 and Vinculin] were selected and validated in ESCC by Western Blot. Furthermore, we performed the QDB and IHC analysis in 64 patients and 117 patients, respectively. The PTMA expression was up-regulated gradually along the progression of ESCC, and the PTMA expression ratio between tumor and adjacent normal tissue was significantly increased along with the progression. Therefore, the PTMA is suggested as a candidate biomarker for ESCC. Our research also presents a new methodological strategy for the identification and validation of novel cancer biomarkers by combining quantitative proteomic with QDB.
